# Early-stage lumbar paraspinal muscle injury: endoscopic versus transforaminal lumbar interbody fusion: a retrospective comparative analysis

**DOI:** 10.1186/s13018-025-06235-8

**Published:** 2025-09-26

**Authors:** Wenbin Xuan, Qinghua Cheng, Yucheng Gao, Ziyi Song, Zengxin Gao

**Affiliations:** 1 Department of Orthopedics, Zhongda Hospital, Southeast University School of Medicine, Dingjiaqiao 87, Nanjing, 210009 China; 2 Nanjing Lishui People’s Hospital, Zhongda Hospital Lishui Branch, Nanjing, 211200 China

**Keywords:** Lumbar spinal stenosis, Paraspinal muscle injury, Endoscopic lumbar interbody fusion, Transforaminal lumbar interbody fusion

## Abstract

**Objective:**

While previous studies frequently reported the clinical efficacy and minimal invasiveness of endoscopic lumbar interbody fusion (Endo-LIF) for lumbar spinal stenosis (LSS), few existing studies quantitatively measure early-stage postoperative paraspinal muscle injury. This study aimed to preliminarily quantify differences in early postoperative paraspinal muscle changes between Endo-LIF and transforaminal lumbar interbody fusion (TLIF) for single-level LSS. The observed alterations, if substantiated in future studies, might offer considerations for tailoring postoperative rehabilitation.

**Methods:**

This retrospective cohort included 90 severe LSS patients undergoing single-level fusion, allocated to Endo-LIF group (*n* = 48) or TLIF group (*n* = 42). Comprehensive data encompassed demographics, operative metrics, patient-reported outcomes (PROs) including Oswestry Disability Index (ODI) and Visual Analogue Scale (VAS), and acute paraspinal muscle trauma biomarkers ((creatine kinase (CK), C-reactive protein (CRP) and erythrocyte sedimentation rate (ESR)). The cross-sectional area (CSA) of paraspinal musculature (multifidus and erector spinae) was quantified at the index instrumented level using axial T2-weighted magnetic resonance imaging (MRI), with measurements obtained preoperatively and during early follow-up (FU). Muscle boundaries were delineated using semi-automated tools (ITK-SNAP v4.0.2) with manual correction, and CSA values were calculated via MATLAB-based custom algorithms.

**Results:**

Preoperative demographics, leg/back pain VAS, ODI, and inflammatory markers were comparable between Endo-LIF and TLIF groups. At 3 days postoperatively, Endo-LIF demonstrated superior back VAS and ODI (*P* < 0.001) but comparable leg pain VAS. Both groups achieved significant PRO improvements (*P* < 0.001). TLIF had significantly higher low back myofascitis incidence (*P* < 0.001). Endo-LIF showed significantly reduced blood loss (58.3[IQR 50, 75] days vs. 214.3[IQR 150, 250] mL) and shorter hospitalization (5.2 [IQR 5, 6] days vs. 7.1 [IQR 7, 8] days) (both *P* < 0.001), but longer operative time and greater fluoroscopy use (*P* < 0.001). Complication rates were similar (*P* = 0.27). CRP and CK levels at postoperative day 1 were significantly higher in TLIF (*P* < 0.001). Long-term follow-up revealed no significant intergroup differences in PROs (all *P* > 0.01). Postoperative paraspinal muscle CSA decreased in both cohorts, with a more pronounced reduction observed in the TLIF group, statistically associated with surgical approach and smoking status (both *P* < 0.001).

**Conclusions:**

Compared with TLIF, Endo-LIF demonstrated relatively early back pain relief, reduced intraoperative blood loss, shorter hospital stays, and lower levels of acute muscle injury markers. These potential benefits were counterbalanced by longer operative durations and greater reliance on fluoroscopy. Both approaches achieved largely comparable long-term functional outcomes with similar safety profiles. Paraspinal muscle CSA measurements suggested a comparatively lesser degree of early muscle injury with Endo-LIF versus TLIF.

## Introduction

The overall incidence of lumbar spinal stenosis (LSS) is gradually increasing as population aging [1]. Lumbar interbody fusion is recommended for patients with severe LSS when conservative measures failed [2]. Transforaminal lumbar interbody fusion (TLIF) was first reported for the treatment of LSS in 1981, with equivalent surgical effects but less intrusion of posterior lumbar vertebrae compared to posterior lumbar interbody fusion (PLIF) [3]. Nevertheless, TLIF is associated with certain disadvantages over affecting posterior structures and the paraspinal musculature [4, 5]. Minimally invasive spinal surgery has gained significant popularity with the increasing clinical application and importance of Enhanced Recovery After Surgery (ERAS) concepts [6]. Endoscopic lumbar interbody fusion (Endo-LIF) was reported first clinically in 2005, facilitated by the improvement of endoscopic techniques [7]. Subsequent studies have expanded evidence on Endo-LIF outcomes, highlighting advantages such as reduced soft tissue dissection, decreased intraoperative bleeding, and accelerated postoperative recovery based on both operative metrics and patient-reported outcomes (PROs) [8].

However, there is a paucity of literature quantitatively quantifying the difference of early-stage paravertebral muscle injury between TLIF and Endo-LIF [9–11]. Therefore, this study preliminarily quantified acute and subacute postoperative paraspinal muscle changes between Endo-LIF and TLIF for single-level LSS. These findings may inform postoperative rehabilitation strategies pending future validation.

## Methods

### Study design

This study was approved by the ethics committee of Southeast University Affiliated Zhongda Hospital (2023ZDSYLL400) in accordance with the Declaration of Helsinki and individual consent for retrospective data collection following strict consecutive patient inclusion was collected. The personal private information of enrolled individuals such as name, job status, home address and medical-record number will not be disclosed or shared with any third parties. Patients underwent MRI scan within 1 months preoperatively and within 6 months postoperatively were considered eligible for this research. Inclusion criteria were: diagnosis of lumbar spinal stenosis without isthmic spondylolisthesis or with degenerative spondylolisthesis ≤ Grade I (Meyerding grade) [1, 12], aged over 50 years, prior ineffective conservative treatment, one level decompression and fusion. Exclusion criteria were: previous spinal surgery, history of tumor and spinal infectious diseases, patients with scoliosis over 10° with expected asymmetric degeneration of spinal muscles, inapplicable for spinal instrumentation due to severe osteoporosis, the value of C-reactive protein (CRP), and erythrocyte sedimentation rate (ESR) above the normal range preoperatively, infective complications including surgical site infection postoperatively. We comprehensively explained the advantages, disadvantages, and indications of both Endo-LIF and TLIF surgical approaches to patients, ensuring full comprehension of both procedures. Patients subsequently autonomously selected one surgical technique and provided written informed consent. The surgical procedures for all included patients were chosen voluntarily by the patients themselves. Although we aimed to minimize selection bias, it still exists and represents one of the limitations of this study.

### Functional evaluation

Demographic data including age, sex, body mass index (BMI), American Society of Anaesthesiologists (ASA) risk score, bone mineral density (BMD), spondylolisthesis, diabetes and smoking status were collected in this research. Back and leg pain Visual Analog Scale (VAS) scores and Oswestry Disability Index (ODI) scores were recorded preoperatively, at 3 days postoperatively, and during follow-up to assess pain severity and functional status. Early follow-up (FU) was defined as postoperative 2 to 6 months, and long-term FU was defined over 1 year. The intraoperative blood loss, the length of operation, intraoperative X-ray exposure times, surgical levels and hospital days (day from surgery to discharge) were collected as surgery-related parameters. Intraoperative blood loss calculation was performed through synchronous multi-modal recording [13]: (1) Calculated by subtracting irrigation fluid volume from total suction output; (2) Estimated via gravimetric analysis of blood-saturated gauzes (1g weight difference = 0.56mL blood).

Serum creatine kinase (CK), CRP and ESR were measured preoperatively and on postoperative day 1 as biomarkers of acute paraspinal muscle trauma following surgical retraction. Sample collection timing was constrained by our institutional clinical protocols: serum inflammatory markers (CK, CRP, ESR) and electrolytes are routinely assessed only at 24 h postoperatively as part of ERAS pathways. This inherent limitation of retrospective studies precluded additional biomarker sampling beyond standard care. The peak level of CK occurs at approximately 24 h after lumbar spinal surgery, this timepoint provides a valid assessment window for acute muscle injury [14, 15].

### Measurements of paraspinal muscle

1.5T MRI with high bandwidth and fast spin-echo scans of the instrumented segments were performed preoperatively and at early FU. Muscular tissue decollement was performed on paraspinal muscle (multifidus and erector spinae) in the procedure of decompression and fusion, no significant injury in psoas muscle was observed following Endo-LIF and TLIF procedures [16, 17]. At the paraspinal region, reliable separation between the erector spinae and multifidus muscles proves challenging, as attempted dissection may introduce substantial measurement error [18]. Therefore, measurement of paraspinal muscle integrity included: Total cross-sectional area of multifidus and erector muscle (TCSA); Functional CSA (FCSA): muscle area isolated from fat or/and edema [19].

To account for anthropometric confounders (height, body shape), relative values were calculated:

$$\text{RTCSA}=\frac{\text{TCSA}}{\text{L}3\text{ superior endplate CSA}},\text{ RFCSA}=\frac{\text{FCSA}}{\text{L}3\text{ superior endplate CSA}}$$

Change in relative muscle area quantified short-term alterations:

$${\Delta RCSA }=\frac{\text{FU}-\text{RFCSA}}{\text{FU}-\text{RTCSA}} - \frac{\text{pre}-\text{RFCSA}}{\text{pre}-\text{RTCSA}}$$

to intuitively demonstrate and assess the changes in paraspinal muscle CSA during short-term FU compared to pre-surgery, where a negative value indicates reduction and a positive value denotes increase in relative muscle area at FU. Average-ΔRCSA represented the mean of measurements from two independent raters.

**Fig. 1:**
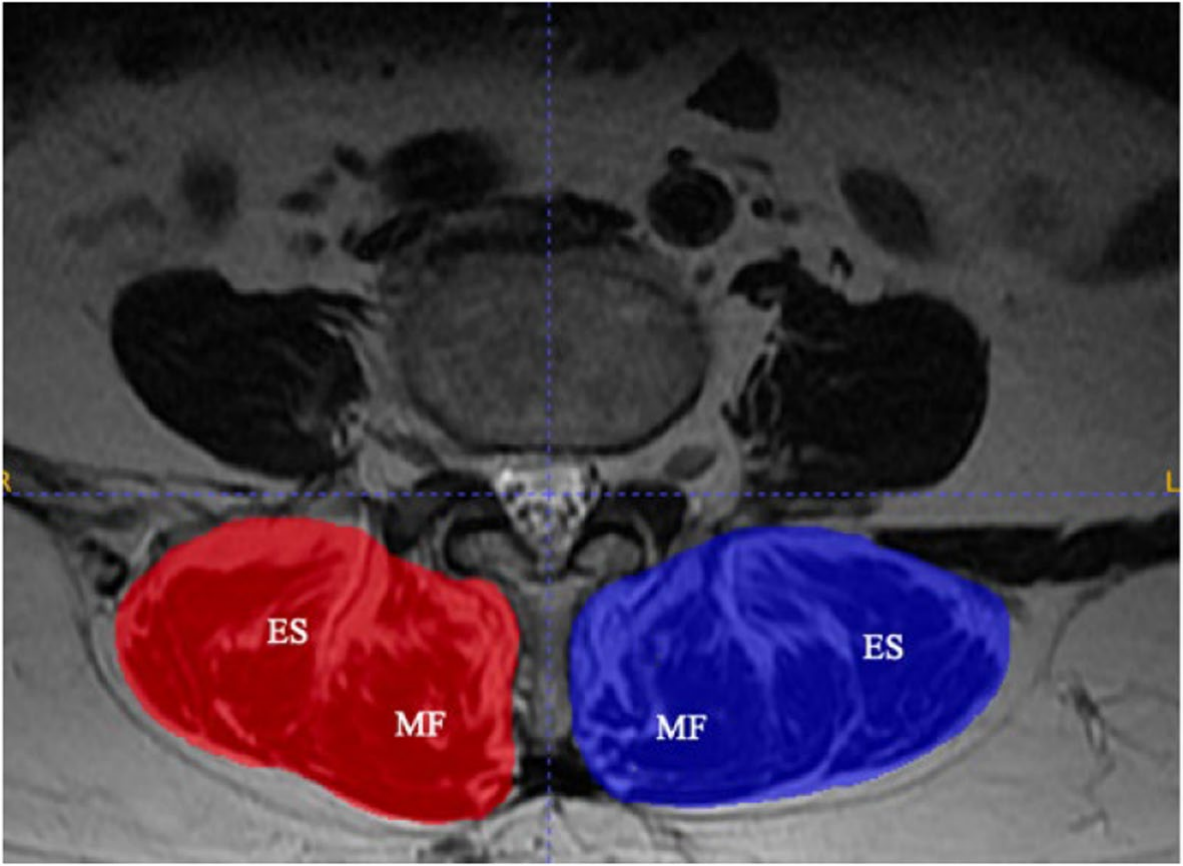
Bilateral manual segmentation of psoas and paraspinal muscles (erector spinae (ES) and multifidus (MF)) on axial T2-weighted MRI

The paraspinal muscle measurements were conducted on axial T2-weighted MRI images at mid-disc level of instrumented segments to minimize implant artifact interference. T2-weighted axial MRI images of enrolled cohorts were downloaded and archived in DICOM format for analysis. Measurements of the paraspinal muscles were conducted using ITK-SNAP (version 4.0.2, www.itksnap.org), a tool for segmenting anatomical structures in medical images. It provides an automatic active contour segmentation pipeline, manually delineate anatomical regions of interest (ROI), along with supporting manual segmentation toolbox [20]. Utilizing ITK-SNAP, 2 ROIs were manually defined and delineated by tracing the fascial border and bony attachment of each muscle component to be measured at each level. Measurements were made for the left and right posterior paraspinal muscles (combined erector spinae and multifidus) on T2-weighted axial images at the at the mid-disc level of instrumented segments (Fig. 1) [21].

**Fig. 2:**
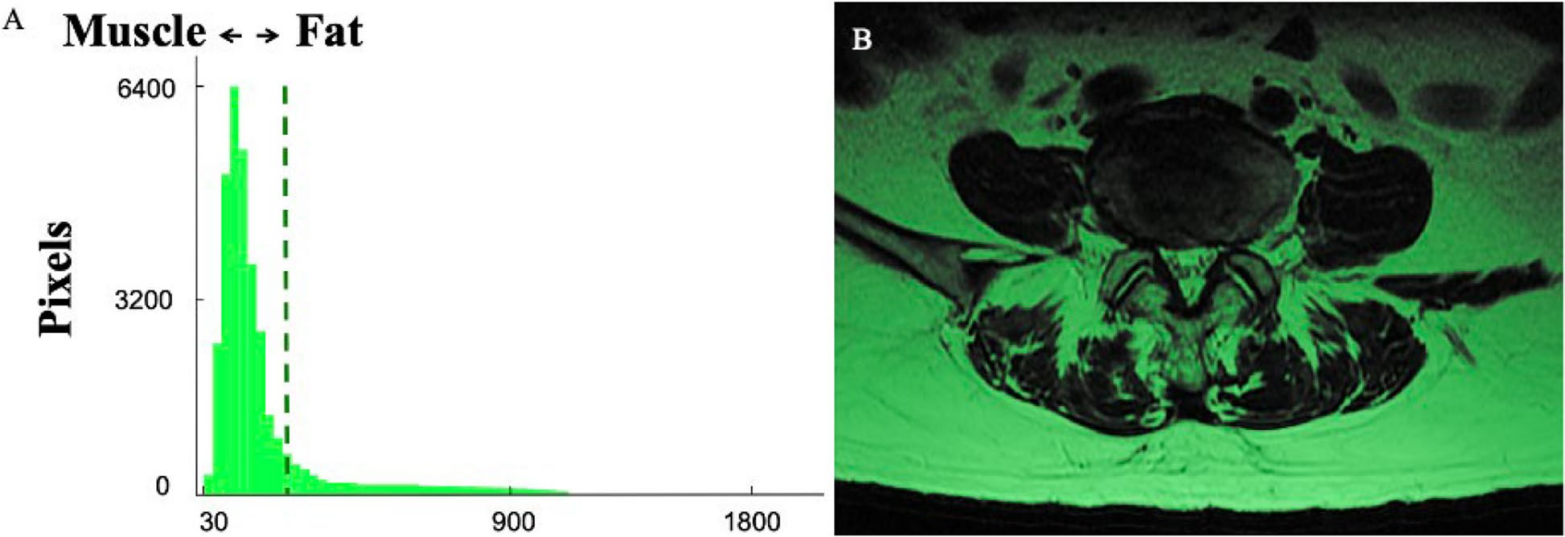
**A** Histogram of pixel intensities within the muscle segmentations used to calculate the automated pixel intensity threshold (fat: to the right of the dotted line; muscle: to the left of the dotted line). **B **Low intensity pixels (shown in dark) were used to calculate the functional cross-sectional area (mm^2^) of paraspinal muscles

A custom-written program on MATLAB (Version R2023a, The MathWorks Inc., USA) was used to calculate an automated pixel intensity threshold incorporating three sequential processes: Intensity bias correction using quadratic polynomial fitting to address field inhomogeneities; Otsu's threshold optimization maximizing inter-class variance to determine the intensity threshold separating fat or and edema (high-intensity pixels) from muscle (low-intensity pixels) [18, 22]; Binary classification generating fat/muscle distribution maps (Fig. 2). The method was established by Nobuyuki Otsu and determines the optimal threshold by minimizing the intra-class variance (the weighted sum of variances of pixel intensities within each class) or equivalently maximizing inter-class variance. By analyzing the histogram of grayscale intensity values, Otsu’s method selects a threshold that best separates two classes, such as lean muscle and fat, based on their statistical distribution, making it a widely used approach in medical imaging for tissue segmentation [22]. The muscle measurement technique for the lumbar spine, as well as the utilization of both software programs, has been previously described and demonstrated excellent inter-rater reliability (ICC > 0.90 in pilot data). The overall sizes (area) of bilateral paraspinal muscles were used for analysis [23].

The muscle measurements were conducted independently by two board-certified spinal neurosurgeons blind to the information of patients mastering in assessing lumbar spinal MRI images to minimize potential errors in muscle margin definition.

### Surgical techniques

Surgical planning comprised MRI, computed tomography (CT), and lumbar spine X-ray in the upright position. All operations were performed under general anesthesia with prone position with knees slightly bent to expand the laminar space. The decompression approach was decided according to the dominant clinical symptoms.

**Fig. 3:**
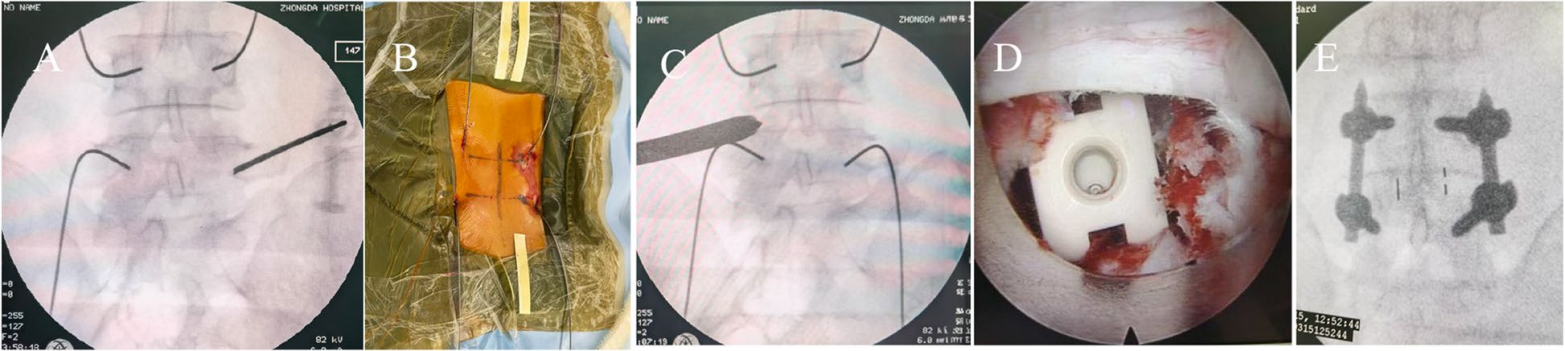
**A** The intraoperative radiograph showed that the skin entry point was located at the pedicle of vertebral arch through fluoroscopic view. **B** The primary needles were replaced by four guide wires and fixed with surgical drape. **C** The radiograph showed the position of the protection cannula during the operation after the working channel was established. **D** The nerve root and cage were checked under the endoscopic visual field. **E** The anterior lumbar spine positions demonstrate the placement of the cage and percutaneous pedicle screw

The inferior articular process, partial superior articular process, vertebra lamina of the approach side and basilar part of spinous process were resected to facilitate cannula insertion under endoscopic visualization. Sufficient decompression of the nerve root was performed with pituitary forceps and radiofrequency. Pituitary forceps and different models of reamer were inserted progressively and performed the discectomy. Next, Pituitary forceps and curettes were used to remove disc and cartilaginous endplates. Cartilaginous endplates were scraped away under endoscopic vision. Then autologous bone of the decompression vertebrae, a suitable PEEK cage (CAPSTONE Spinal System, USA) filled with allogeneic bone (BIO-GENE, Beijing, China) were inserted into the disk space sequentially (Fig. 3). Percutaneous pedicle screw fixation was conducted after removing the endoscopy and working tubes. Bilateral “over the top” decompression through unilateral approach was applied when patients showed symptoms in both lower extremities [24, 25].

All Endo-LIF procedures were performed by a senior surgeon Dr. Gao who had mastered the technique via cadaveric training and proctored surgeries in Oct 2019.Our center served as a regional Endo-LIF training Hub under the guidance of Dr. Gao. We further mitigated learning curve impacts by excluding the first 30 Endo-LIF cases prior to study initiation.

The TLIF approach involved a midline incision, allowing access to the disc space suitable for intended levels. The spinal canal was entered via a unilateral laminectomy and inferior facetectomy, which facilitated bone graft placement. Targeted discs and cartilaginous endplates were removed, then the PEEK cage (CAPSTONE Spinal System, USA) filled with morselized bone were placed into the disk space. Fixed and sutured layered operation incision [26].

A standardized postoperative rehabilitation protocol, including progressive exercise regimens, was implemented for all patients in both groups.

### Statistical Analysis

Data were entered, sorted and partially analyzed using Microsoft Excel 2016 (Microsoft Corporation, United States). Proportions were used for categorial variables to summarize their distribution, and comparisons between categorial variables were performed utilizing the Pearson χ^2^ test or Fisher's exact test when any expected frequency < 5. The Shapiro–Wilk test was used to assess for normal distribution of continuous variables. Descriptive statistics were performed using calculation of mean ± standard deviations (SD) for normally distributed data, median [interquartile range (IQR)] for abnormally distributed continuous data. Comparisons of continuous variables were performed according to their normal distribution utilizing the independent t-test; and the Mann–Whitney U test for non-normally distributed data. For the inter-rater reliability between two raters, the intraclass correlation coefficient (ICC) and its 95% confidence interval (CI) were calculated using a mean rating (k = 2), an absolute agreement definition, and a 2-way mixed-effects model. Reliability was interpreted as follows: ICC > 0.90 excellent, 0.80 to 0.90 good, 0.70 to 0.80 acceptable, and < 0.70 poor [18]. Multivariable linear regression models were used to assess the association between surgical approach and paraspinal muscle parameters, adjusted for age, sex, BMI, BMD, smoking, ASA score, surgery level, spondylolisthesis and ΔRCSA. The data was statistically analyzed with the SPSS 26.0 (IBM Corp., USA). The statistical significance was set as *P* < 0.01.

## Results

### Clinical outcomes

Ninety patients who underwent single-level lumbar interbody fusion between Oct 2020 and Jan 2024 in the spine surgery department of Zhongda Hospital were retrospectively analyzed, including 48 patients in Endo-LIF group (24 male, 24 female, mean age 67.2 ± 7.7 years, range 55–88) and 42 in TLIF group (19 male, 23 female, average age 66.1 ± 9.9 years, range 51–87) (Table 1). There was no intergroup statistical significance between two groups in baseline demographics, including age, sex, BMI, etc. (all *P* > 0.01, Table 1).

**Table 1:** Demographic and clinical features of patients

Variables	Endo-LIF group	TLIF group	χ^2^/t/Z values	*P* value
Sex (M/F)	24/24	19/23	χ^2^ = 0.204	0.79
Age (year)	67.2 ± 7.7	66.1 ± 9.9	t = 0.597	0.55
BMI (kg/m^2^)	24.2 ± 2.0	23.9 ± 2.0	t = 1.004	0.32
Osteoporosis (T score ≤ −2.5)	3/48	3/42	χ^2^ = 0.029	0.32(Fisher’s Exact Test)
ASA scores				
1	21/48	17/42	χ^2^ = 0.156 Cramer's V = 0.038	0.84 (Fisher’s Exact Test)
2	22/48	21/42		
3	5/48	4/42		
Diabetes	12.5% (6/48)	14.3% (6/42)	χ^2^ = 0.062	0.82
Smoking	35.4% (17/48)	31.0% (13/42)	χ^2^ = 0.201	0.23
Spondylolisthesis	31.3% (15/48)	38.1% (16/42)	χ^2^ = 0.465	0.11

Data are shown as mean ± standard deviation for normally distributed continuous variables

*P* value indicates the difference between patients in Endo-LIF group and TLIF group. *Endo-LIF* endoscopic lumbar interbody fusion, *TLIF* transforaminal lumbar interbody fusion, *M* male, *F* female, *FU* follow-up, *BMI* body mass index, *ASA* American Society of Anaesthesiologists

Early follow-up (FU) durations were comparable between groups: Endo-LIF (median 3.2 months [IQR 3, 4]) vs TLIF (median 3.3 months [IQR 3, 4]). PROs including leg pain VAS, back pain VAS and ODI demonstrated no intergroup differences preoperatively (all *P* > 0.01). At postoperative day 3, the Endo-LIF group demonstrated significantly superior improvements in back pain VAS (*P* < 0.001) and ODI (*P* < 0.001) versus TLIF, while leg pain VAS differences remained statistically non-significant (*P* = 0.04). Both groups achieved clinically significant PRO improvements at early FU versus baseline (all *P* < 0.001). Low back myofascitis incidence was significantly higher in the TLIF group versus Endo-LIF (*P* < 0.001; Table 2).

**Table 2:** Comparison of short-term clinical outcomes between both groups

Variables	Endo-LIF group	TLIF group	χ^2^/t/Z values	*P* value
FU (month)	3.2 [IQR 3, 4]	3.3 [IQR 3, 4]	Z = −0.803	0.65
VAS (back)				
Pre-surgery	6.1 [IQR 5, 7]	6.0 [IQR 5, 6.25]	Z = −0.085	0.97
3-day post-surgery	4.3 [IQR 3.25, 5]	5.3 [IQR 5, 6]	Z = −3.976	< 0.001*
Short-term FU	1.4 [IQR 1, 2]	1.8 [IQR 1, 3]	Z = −1.731	0.08
VAS (leg)				
Pre-surgery	6.8 [IQR 6, 8]	6.9 [IQR 6, 8]	Z = −0.074	0.81
3-day post-surgery	4.2 [IQR 3, 5]	4.9 [IQR 4, 6]	Z = −2.092	0.04
Short-term FU	1.2 [IQR 0.25, 2]	1.4 [IQR 0, 2.25]	Z = −0.789	0.38
ODI				
Pre-surgery	55.7 ± 6.8	55.1 ± 7.5	t = 0.406	0.69
3-day post-surgery	39.1 ± 6.6	47.9 ± 4.7	t = −7.188	< 0.001*
Short-term FU	21.7 ± 2.6	21.8 ± 2.4	t = −0.062	0.95
Ratio of myofascitis				
Pre-surgery	54.2% (26/48)	54.8% (23/42)	χ^2^ = 0.033	0.96
Short-term FU	35.4% (17/48)	59.6% (25/42)	χ^2^ = 5.230	< 0.001*

Data labeled* means statistical significance. *VAS* Visual Analog Scale, *ODI* Oswestry Disability Index

Sixty-four of the 90 patients completed long-term FU through phone interviews conducted by a data coordinator not involved with clinical care. Participants lost to long-term FU were due to: Loss of contact from phone number changes (*n* = 13 Endo-LIF, *n* = 13 TLIF); Non-study-related comorbidities (Endo-LIF: 1). During the telephone FU, an 83-year-old female patient in Endo-LIF group reported experiencing severe back pain over the past month that had been severely affecting her daily life (ODI score 78). She had not attended her scheduled FU visit at our institution. Her local physician suspected possible osteoporotic fracture of thoracic vertebra as the cause. Given the patient's special circumstances of unrelated etiology, she was excluded from subsequent analyses. Attrition rates (29.2% Endo-LIF vs. 31.0% TLIF, χ^2^ = 0.12, *P* = 0.73) did not differ significantly between groups. The final analytical cohort with long-term PROs consisted of 35 patients in Endo-LIF group (16 male, 19 female, mean age 64.6 ± 6.0 years) and 29 in TLIF group (15 male, 14 female, average age 61.6 ± 7.6 years) (Table 3). The mean early FU was 28.9 ± 11.2 months, 33.6 ± 8.2 months, in Endo-LIF and TLIF groups respectively. Among patients with long-term FU, VAS for leg and ODI scores demonstrated no statistically significant differences between Endo-LIF and TLIF group across assessed periods: preoperative, 3-day post-operation, short-term FU, and long-term FU (all *P* > 0.01; Table 3). While a trend toward reduced leg pain was observed with Endo-LIF at 3 days (VAS 4.2 [IQR 3, 5] vs. 4.9 [IQR 4, 6], *P* = 0.05), this difference approached but did not exceed conventional statistical significance thresholds. The ODI scores and low back VAS scores in Endo-TLIF group 3 days post-operation were significantly lower than that in the TLIF group (both *P* < 0.001).

**Table 3:** Comparison of PROs between two groups of patients with long-term FU

Variables	Endo-LIF group	TLIF group	χ^2^/t/Z values	*P* value
Sex (M/F)	16/18	15/14	χ^2^ = 0.204	0.46
Age(year)	64.6 ± 6.0	61.6 ± 7.6	t = −0.616	0.11
Final-FU (month)	28.9 ± 11.2	33.6 ± 8.2	t = −1.87	0.06
VAS (back)				
Pre-surgery	6.1 [IQR 5, 7]	6.0 [IQR 5, 6]	Z = −0.413	0.68
3-day post-operation	4.4 [IQR 4, 5]	5.5 [IQR 5, 6]	Z = −3.338	< 0.001*
Short-term FU	1.4 [IQR 1, 2]	1.8 [IQR 2, 3]	Z = −1.529	0.13
Final FU	1.0 [IQR 1, 1]	0.9 [IQR 0, 1]	Z = −0.209	0.83
VAS (leg)				
Pre-surgery	6.9 [IQR 6, 8]	6.6 [IQR 6, 7.5]	Z = −0.825	0.41
3-day post-operation	4.2 [IQR 3, 5]	4.9 [IQR 4, 6]	Z = −1.984	0.05
Short-term FU	1.4 [IQR 1, 2]	1.5 [IQR 0, 2]	Z = −0.207	0.84
Final FU	1.0 [IQR 0, 2]	0.9 [IQR 0, 1.5]	Z = −0.372	0.71
ODI				
Pre-surgery	55.7 ± 6.8	55.1 ± 7.5	t = −0.173	0.86
3-day post-operation	38.7 ± 6.4	48.0 ± 4.5	t = −5.036	< 0.001*
Short-term FU	22.5 ± 2.2	22.2 ± 2.4	t = −0.308	0.76
Final FU	12.4 ± 3.1	13.2 ± 3.2	t = −1.228	0.22

Data labeled* means statistical significance

### Surgery-related outcomes

**Fig. 4:**
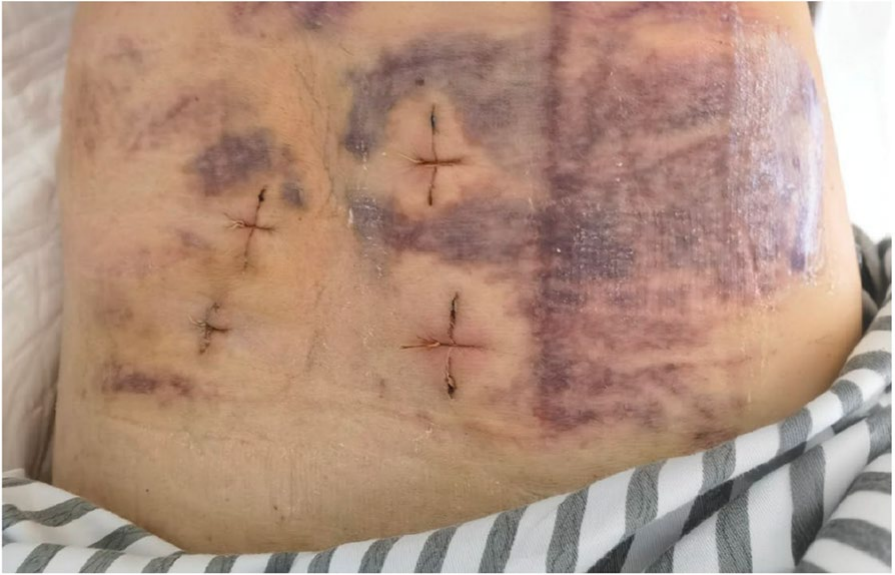
Subcutaneous ecchymoses occurred in one 59-year-old female patient

This research retrospectively analyzed patients who received one level decompression and fusion, all these levels were at L4/5 and L5/S1 (Table 4). Hospitalization length was defined as the duration starting from surgery to discharge day. Compared with TLIF, Endo-LIF demonstrated: Reduced blood loss (median 58.3 mL [IQR 50, 75] vs 214.3 mL [IQR 150, 250]; *P* < 0.001); Shorter hospitalization (median 5.2 days [IQR 5, 6] vs 7.1 days [IQR 7, 8]; *P* < 0.001); Longer operative time (mean 190.7 ± 7.8 vs 113.5 ± 6.8 min; *P* < 0.001); Higher fluoroscopy frequency (median 38.6 [IQR 37, 40] vs 3.1 [IQR 3, 4]; *P* < 0.001). Postoperative subcutaneous ecchymoses occurred in two cases of Endo-LIF group, presumably attributable to the absence of drain placement coupled with suboptimal hemostatic control during surgery (Fig. 4). Gluteal epithelial neuritis related pain in the upper posterior thigh was observed in one patient of Endo-LIF group and diminished after hot compress within 5 days of surgery. In the TLIF group, there was one case of cerebrospinal fluid (CSF) leakage, which recovered after conservative treatment within 7 postoperative days. One patient in TLIF group experienced numbness in the contralateral lower limb postoperatively, the patient reported significant improvement 2 weeks after surgery, which might be due to intraoperative nerve root irritation. Suboptimal screw placement was identified postoperatively in one subject of TLIF group, but as the patient showed no neurological symptoms, no further intervention was undertaken. Cage subsidence rates were comparable (Endo-LIF 3/48 vs TLIF 3/42) [27] (Table 4).

**Table 4:** Surgery-related results of Endo-LIF group and TLIF group

	Endo-LIF group	TLIF group	χ^2^/t/Z values	*P* value
Level fused				
L4/5 (M/F)	14/12	11/12	χ^2^ = 0.177	0.69
L5/S1 (M/F)	10/12	8/11	χ^2^ = 0.046	0.57
Surgery time (min)	190.7 ± 7.8	113.5 ± 6.8	t = 49.919	< 0.001*
Fluoroscopy times	38.6[IQR 37, 40]	3.1[IQR 3, 4]	Z = −8.465	< 0.001*
Blood loss(mL)	58.3[IQR 50, 75]	214.3[IQR 150, 250]	Z = −8.557	< 0.001*
Hospital length (day)	5.2[IQR 5, 6]	7.1[IQR 7, 8]	Z = −8.584	< 0.001*
Complications	12.5% (6/48)	14.3% (6/42)	χ^2^ = 0.315	0.27
Subcutaneous ecchymosis	4.2% (2/48)	0 (0/42)		
Neurological symptom	2.1% (1/48)	2.4% (1/42)		
CSF leak	0 (0/48)	2.4% (1/42)		
Screw malposition	0(0/48)	2.4% (1/42)		
Cage subsidence	6.3% (3/48)	7.1% (3/42)		

Data labeled* means statistical significance. *P* value indicates the difference between patients in Endo-LIF group and TLIF group. *CSF* cerebrospinal fluid

### Laboratory results

ESR, CRP, and CK served as biomarkers of acute intraoperative paraspinal muscle trauma. Preoperative levels showed no significant intergroup differences (CK, *P* = 0.83; ESR, *P* = 0.90; CRP, *P* = 0.83) (Table 5). By postoperative day 1, all biomarkers increased significantly from preoperative baselines (all *P* < 0.001). At postoperative day 1: ESR remained comparable between groups (*P* = 0.06); CRP and CK were significantly elevated in the TLIF group versus Endo-LIF (both *P* < 0.001), suggesting greater acute muscle trauma with TLIF.

**Table 5:** Laboratory results of Endo-LIF group and TLIF group

	Endo-LIF group	TLIF group	t value	*P* value
CK (U/L)				
Pre-op	76.9 ± 12.2	77.4 ± 11.5	−0.221	0.83
Post-op	319.2 ± 52.5	388.5 ± 58.8	−5.910	< 0.001*
ESR (mm/h)				
Pre-op	7.5 ± 3.5	7.6 ± 3.9	−0.127	0.90
Post-op	27.6 ± 7.9	30.6 ± 6.7	−1.932	0.06
CRP (mg/L)				
Pre-op	1.6 ± 0.9	1.6 ± 0.8	−0.215	0.83
Post-op	24.0 ± 4.6	29.8 ± 4.9	−5.781	< 0.001*

Data labeled* means statistical significance. *Pre-op* preoperative, *Post-op* one day post-operation, *CK* creatine kinase, *CRP* C-reactive protein, *ESR* erythrocyte sedimentation rate

### Radiological parameters

**Fig. 5:**
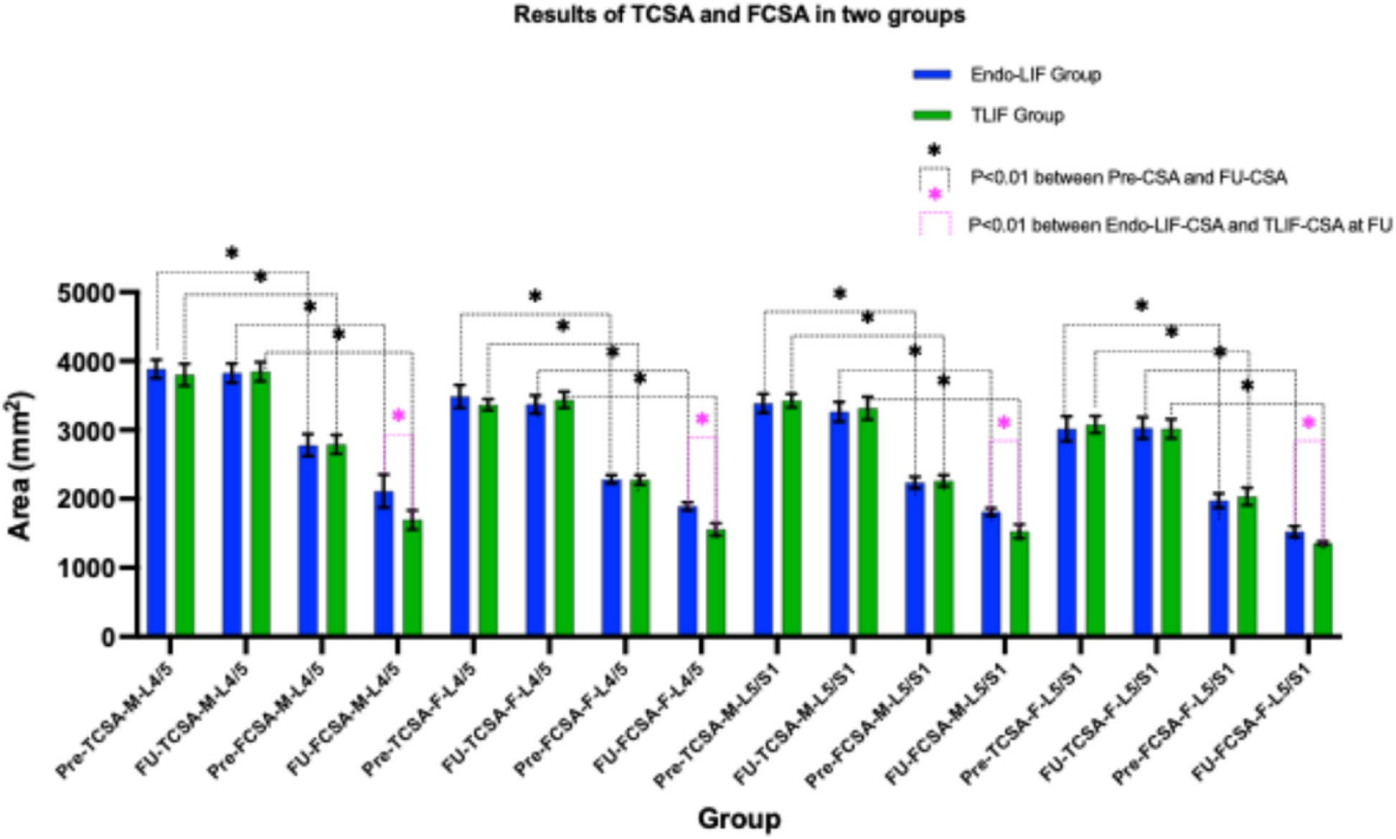
Shows TCSA and FCSA in male and female patients at L4/5, L5/S1. Pre-TCSA-L4/5 indicates preoperative TCSA of male patients at L4/5 level group; FU -TCSA-L4/5 means TCSA of male patients at L4/5 at FU. M, male; F, female; Pre, preoperative; FU, follow up; TCSA, total cross-sectional area of paraspinal muscle; FCSA, functional cross-sectional area of paraspinal muscle

When stratified by spinal level and gender, preoperative CSA showed no significant intergroup differences between Endo-LIF and TLIF cohorts (all *P* > 0.01). Preoperative CSA was higher than CSA at FU at stratified levels (*P* < 0.01), with L4/5 CSA greater than L5/S1 CSA preoperatively and at FU (*P* < 0.01). The Endo-LIF group demonstrated higher CSA at FU versus TLIF (*P* < 0.01), while males exhibited larger CSA than females at matched spinal levels and equivalent operative timepoints (*P* < 0.01) (Fig. 5).

### Multivariable linear regression analysis results

**Fig. 6:**
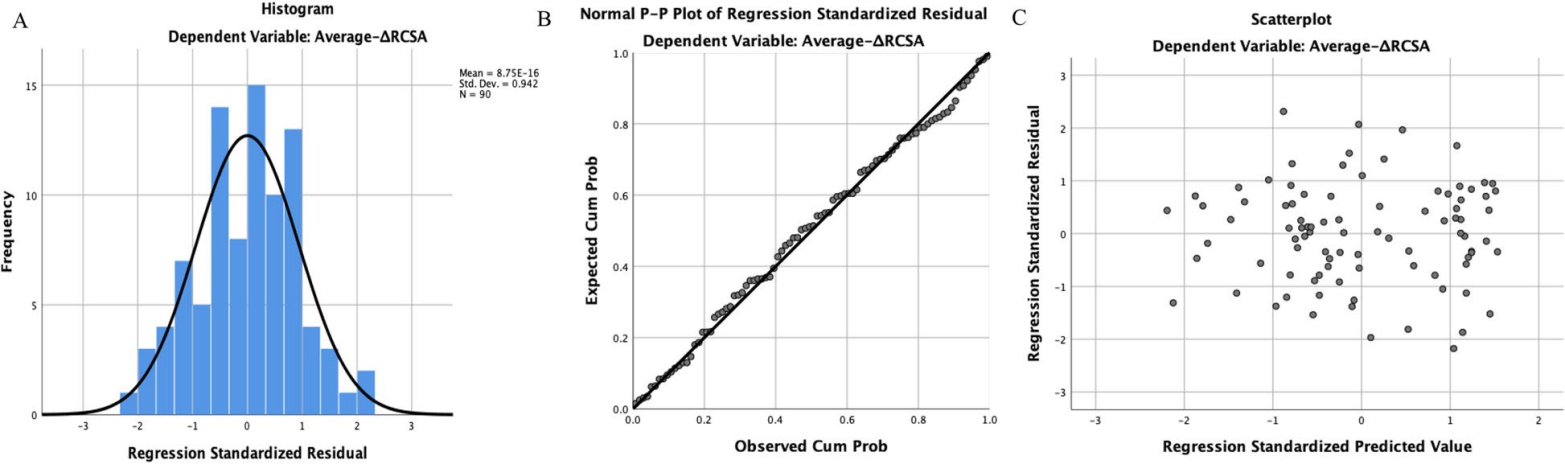
**A-C** Histogram, Normal P-P plot and Scatter plot of standardized residuals versus standardized predicted values of dependent variable

The histogram, Normal P-P plot, and Scatter plot of standardized residuals versus standardized predicted values indicate that the points are distributed relatively evenly, with residuals approximating a normal distribution (Fig. 6). All variance inflation factor (VIF) values were less than 5, suggesting no multicollinearity among the independent variables.

Adjusting for the confounders age, sex, BMI, fused-segment, BMD, ASA score, diabetes, smoking and spondylolisthesis, surgery approach showed a positive estimate of −0.160 (95% CI [−0.193, −0.127], < 0.001) while it was −0.105 for smoking (95% CI [−0.151, −0.059]) (Table 6). These results indicate that TLIF procedures might reduce about 16.0% of RCSA of paraspinal muscle in short-term FU, smoking could decrease 10.5% of RCSA of paraspinal muscle in short-term FU. There was no statistically significance between ΔRCSA and age (*P* = 0.209), sex (*P* = 0.213), BMI (*P* = 0.223), fused segment (*P* = 0.621), BMD (*P* = 0.525), ASA score (*P* = 0.600), diabetes (*P* = 0.236) and spondylolisthesis (*P* = 0.535).

**Table 6:** Results of multivariable linear models for the prediction of Average-ΔRCSA correcting for the confounders

Metrics	Unstandardized Coefficients Beta	Standardized Coefficients Beta	t value	*P* value	95% CI [Lower, Upper]	Tolerance	VIF
Group	−0.160	−0.702	−9.754	< 0.001*	[−0.193, −0.127]	0.933	1.072
Age	−0.002	−0.132	−1.266	0.209	[−0.004, 0.001]	0.446	2.242
Sex	−0.030	−0.132	−1.256	0.213	[−0.078, 0.018]	0.435	2.299
BMI	−0.007	−0.089	−1.229	0.223	[−0.018, 0.004]	0.926	1.079
Fused-segment	0.008	0.036	0.497	0.621	[−0.025, 0.041]	0.932	1.072
BMD	−0.007	−0.075	−0.639	0.525	[−0.029, 0.015]	0.355	2.818
ASA score	−0.007	−0.040	−0.527	0.600	[−0.034, 0.020]	0.818	1.222
Spondylolisthesis	0.011	0.046	0.623	0.535	[−0.023, 0.044]	0.895	1.117
Diabetes	−0.027	−0.087	−1.195	0.236	[−0.071, 0.018]	0.911	1.098
Smoking	−0.105	−0.429	−4.565	< 0.001*	[−0.151, −0.059]	0.547	1.827

Data labeled* means statistical significance. *VIF* variance inflation factor

### Inter-observer reliability for the radiological measurements

The inter-observer analysis of the CSA from 90 enrolled patients was performed, revealing excellent agreement regarding all the preoperative CSA, good agreement regarding all the preoperative CSA, between the two independent observers regarding all the measured radiographic parameters (Table 7).

**Table 7:** Inter-observer reliability for the CSA measurements

	ICC	95% CI [Lower, Upper]	F test with true value 0	
			Value	Sig (*P* Value)
Pre-TCSA in Endo-LIF group	0.932	[0.897, 0.956]	525.105	< 0.001*
Pre-FCSA in Endo-LIF group	0.967	[0.945, 0.981]	457.772	< 0.001*
FU-TCSA in Endo-LIF group	0.898	[0.852, 0.931]	544.163	< 0.001*
FU-FCSA in Endo-LIF group	0.855	[0.792, 0.901]	410.457	< 0.001*
Pre-TCSA in TLIF group	0.965	[0.942, 0.979]	528.407	< 0.001*
Pre-FCSA in TLIF group	0.943	[0.912, 0.964]	463.381	< 0.001*
FU-TCSA in TLIF group	0.869	[0.812, 0.911]	573.243	< 0.001*
FU-FCSA in TLIF group	0.842	[0.778, 0.889]	389.322	< 0.001*

Data labeled* means statistical significance

## Discussion

### Background and objective

PLIF was first introduced for lumbar disc diseases in 1943 by Cloward, followed by improved procedures such as anterior lumbar interbody fusion (ALIF), TLIF, etc. [28, 29]. TLIF preserved posterior midline structures and reduced the retraction of nerve roots during operative procedures compared to PLIF, most importantly, the technique could be performed as a minimally invasive procedure [30]. Subsequently, Endo-LIF was introduced and extensively employed for the purposes of sufficient decompression with minimization of operation-related trauma and its sequelae as surgical techniques and endoscopic devices improved [6, 31]. Lumbar paraspinal muscles play a critical biomechanical role in stabilizing the spinal column during dynamic movements such as flexion–extension and weight-bearing activities. These muscles function as primary dynamic stabilizers for facilitating both mobility and stability during functional tasks [32]. Dissection and traction are usually performed on the lumbar paraspinal muscle (especially MF) during PLIF according to Michael et al. in a prospective randomized clinical trial study [33]. Conventional open approaches induce iatrogenic muscle damage through two primary mechanisms: Structural disorganization from direct muscle dissection and myofibrillar disruption during retractor placement; Vascular injury and nerve transection during surgical procedures, triggering atrophy via combined ischemic and neurogenic pathways [34, 35]. Consequently, objective quantification of paraspinal muscle surgical trauma warrants comparative analysis between Endo-LIF and TLIF techniques.

### Patient-reported health status results

The observed differences between Endo-LIF and TLIF suggest potentially important clinical distinctions. Notably, Endo-LIF was associated with improved early postoperative pain control. At 3 days, Endo-LIF patients reported lower back pain scores (median VAS 4.3 [IQR 3.25, 5] vs. 5.3 [IQR 5, 6]; *P* < 0.001) and lower disability scores (mean ODI 39.1 ± 6.6 vs. 47.9 ± 4.7; *P* < 0.001) compared to TLIF. This earlier pain reduction may contribute to observed trends of lower opioid utilization and earlier time to ambulation, factors relevant to postoperative recovery. Endo-LIF demonstrated a significantly lower incidence of chronic myofascitis (35.4% [17/48] vs. 59.6% [25/42]; *P* < 0.001). This reduction in chronic myofascitis, potentially linked to Endo-LIF's less invasive approach and reduced iatrogenic muscle trauma, might be particularly relevant for patients with high physical demands to help preserve paraspinal function. Comparative analysis revealed no statistically significant intergroup differences in ODI or VAS scores at preoperative baseline, early postoperative FU and final FU assessment. Longitudinal assessment demonstrated clinically statistically significant improvements in both functional and pain metrics at early and final FU intervals relative to preoperative status. This suggests that both surgical techniques yielded comparable clinical outcomes, a finding consistent with prior network meta-analysis evidence [36].

In the assessment of postoperative lower extremity pain, VAS scores on postoperative day 3 demonstrated no statistically significant intergroup differences. This null finding might be attributable to limited patient ambulation during the acute postoperative phase, potentially obscuring functional pain differentials. The borderline significance of early leg pain reduction (*P* = 0.05) warrants caution in interpretation. This finding should be considered exploratory, as it may reflect limited statistical power rather than a definitive clinical effect. Future studies with larger cohorts are needed to validate this observation. Conversely, significant intergroup disparities were observed in both lumbar pain VAS scores ODI scores at the 3-day post-operation timepoint (both *P* < 0.01). Several factors may explain this divergence: Firstly, the Endo-LIF procedure resulted in significantly reduced iatrogenic injury to lumbar soft tissues (including skin, fascia, and musculature) compared to the TLIF approach. Additionally, the smaller incision associated with Endo-LIF may have positively influenced pain perception. Thirdly, the routine use of closed-suction drains in the TLIF cohort may have contributed to increased patient discomfort. Furthermore, earlier ambulation in the Endo-LIF group likely mitigated lower back pain associated with prolonged bed rest and musculoskeletal deconditioning. Early pain reduction following Endo-LIF might facilitate accelerated postoperative ambulation. However, a limitation of this study is the lack of sufficient short-term postoperative data to determine the comparative efficacy of the two surgical approaches in PROs during the initial recovery phase. Return to work (RTW), encompassing both the duration of occupational absence and residual work capacity limitations following spine surgery, represents an important functional outcome variable that warrants standardized integration in our future clinical investigations [37].

### Surgery-related outcomes and complications

Comparative analysis revealed significantly prolonged mean operative time and increased fluoroscopy exposure in the Endo-LIF cohort. Conversely, the Endo-LIF approach demonstrated advantageous reductions in both blood loss and length of hospital stay, which was consistent with previous meta-analysis study [36]. The shorter duration of hospitalization might contribute to lower costs. In the Endo-LIF cohort, two cases of subcutaneous ecchymosis were observed, attributable to hematoma migration to subcutaneous planes secondary to the absence of surgical drainage. No permanent neurological injuries occurred in either group: One instance of gluteal epithelial neuritis was documented in Endo-LIF group, likely resulting from intraoperative hypothermia and prolonged operative duration; One patient in TLIF group developed transient contralateral lower limb paresthesia that resolved spontaneously within 7 days, which we reckon might attribute to nerve root irritation during surgical manipulation. One CSF leakage occurred in the TLIF group. Short-term FU revealed three cases of cage subsidence in each cohort.

As an established minimally invasive alternative, minimally invasive TLIF (MIS-TLIF) demonstrates comparable one-year and two-year outcomes to Endo-LIF per existing literature [38, 39]. Our protocol originally planned a retrospective comparative analysis using MIS-TLIF as the control intervention for Endo-LIF. However, Hospital Registry Data from our tertiary medical center (2020–2024) in Jiangsu Province indicated that MIS-TLIF constituted < 5% of lumbar fusion procedures, reflecting real-world practice patterns at this institution. While this limits direct comparison of minimally invasive techniques, it provides benchmark data against conventional standards. Endo-LIF uniquely accesses Kambin's triangle without osseous channel creation, preserving facet joints, which minimizes procedural complexity, iatrogenic nerve injury, and instability risks. However, Endo-LIF technique necessitates increased intraoperative fluoroscopy utilization for incision localization and cage placement verification, resulting in significantly higher radiation exposure compared to TLIF. Furthermore, Endo-LIF presents substantial technical challenges characterized by a steep learning curve, wherein surgeon proficiency directly influences postoperative recovery trajectories and complication profiles [40]. According to Guo et al., surgical proficiency in Endo-LIF necessitates traversing a learning curve of no fewer than 29 cases [41]. Therefore, all procedures included in this study occurring after the 30-case learning curve threshold (Jan 2020 to Oct 2020) to mitigate the impact of initial surgical inexperience on outcomes. Furthermore, to explicitly evaluate learning curve effects, we stratified Endo-LIF cases into: early cohort, first 24 cases (Oct 2020-Jun 2022), late cohort, subsequent 24 cases (Jul 2022-Jan 2024). No statistically significant difference (*P* = 0.41) was observed in surgery time (normally distributed) between early cohort (191.7 ± 8.3 min) and late cohort (189.8 ± 7.3 min); in blood loss (nonnormal distribution, *P* = 0.13) between early cohort (50 mL [IQR 50, 100]) and late cohort (50 mL [IQR 50, 100]). Restricted decompression capabilities of Endo-LIF under endoscopic visualization contraindicate its application in high-grade spondylolisthesis (Meyerding ≥ III) and complex foraminal stenosis, where macroscopic neural element liberation is essential [17]. Collectively, these technical constraints restrict the clinical adoption of Endo-LIF. While we acknowledge that TLIF represents a more invasive historical control, it was selected as the comparator for two reasons: Firstly, TLIF remains the most prevalent surgical standard in our institution's practice (> 80% of primary cases during study design). Secondly, it provides the clearest contrast for evaluating novel access-related benefits (e.g., tubular dilation vs. muscle stripping) [36].

### Laboratory parameters

In sports practice, the degree of muscle cell injury is usually reflected by indirect indicators of skeletal muscle injury due to the limitation of muscle biopsy [42]. Therefore, proteins related to inflammatory reaction are introduced to evaluate the injury to skeletal muscle, of which CK has been put into application extensively [43]. The CK levels on postoperative days 1 in TLIF group was significantly greater than that in the other group, indicating more severe muscular structure damage in TLIF group. The results were consistent with previous studies [10, 16]. ESR and CRP have been used as an index for evaluating inflammatory response, which were introduced to estimate the inflammatory responses caused by surgical procedure in this study. ESR and CRP showed higher level 1 day post-operatively in both groups than those of pre-operatively. There was no statistical significance in relation to ESR between Endo-LIF group and TLIF group before and after surgery, which might be due to the following two reasons: Exudation from the surgical site tissue could result in increase of ESR as drainage tubes were not used in patients undergoing Endo-LIF surgery. Otherwise, the time taken to reach the peak level of ESR in patients of two groups might differ. The observed advantages in muscle biomarkers and blood loss demonstrated reduced approach-related morbidity versus open surgery, but do not imply inherent superiority over other minimally invasive techniques. Moreover, the absence of serial daily monitoring for laboratory biomarkers (CK ESR and SRP), a critical methodological limitation of this study, substantially compromises the validity of postoperative recovery assessments.

### Radiological assessment results

Lumbar fasciitis is considered as one common factor leading to low back pain, which is induced by inflammatory aseptic processes and/or microinjuries to the myofascial [44]. Patients with degenerative lumbar diseases are prone to suffer from fasciitis as ligament laxity, muscle fatigue, muscle degeneration and fat infiltration occurs with aging [45]. The results in this study indicated patients underwent Endo-LIF procedure suffered less myofascitis than patients underwent TLIF processes at early follow up (*P* < 0.01), which might be due to the following reasons: less damage of skin and muscular tissue during Endo-LIF surgical procedure, easier revascularization of soft tissue in surgical site, and early ambulation for mobilization, etc. Reduced incidence of postoperative lumbar fasciitis facilitates early ambulation, which plays an important role in helping patients return to daily activities.

Paraspinal muscles, consisting of the bilateral multifidus and erector spinae, play a significant role in the development and prognosis of lumbar degenerative disorders [46, 47]. Furthermore, paraspinal muscle volume is considered to be an independent factor influencing screw loosening and cage subsidence [18, 48]. This investigation sought to characterize differential early paraspinal muscle injury patterns between Endo-LIF and conventional TLIF approaches through multimodal quantitative assessment. The RTCSA and RFCSA were calculated as normalization ratios: relative functional and total CSA divided by the L3 superior endplate surface area, mitigating confounding from vertebral morphometric variability such as body shape and height [49]. Importantly, the early postoperative reduction in FCSA may partly reflect reversible inflammatory processes, edema for example, rather than permanent muscle degeneration. Edema increases proton density and T2 relaxation time, potentially exaggerating the apparent reduction in functional muscle area on fat-suppressed T2-weighted sequences. This could explain the dissociation between stable TCSA and reduced FCSA in the acute phase. The exclusive focus on early-stage muscle injury (≤ 6 months post-operatively) precluded comprehensive assessment of long-term degeneration patterns. This limitation stems primarily from clinical realities: elective MRI beyond one year was obtained in only approximately 10% of the cohort, largely attributable to cost constraints and institutional follow-up protocols. Future investigations should incorporate structured longitudinal MRI protocols, including scheduled examinations at 2- and 5-year intervals, to characterize the temporal progression of fatty infiltration. Moreover, muscle quality measures such as signal intensity normalization was not systematically applied. This limitation impedes accurate quantification of pathological changes including edema, fat infiltration, and fibrosis, which are critical indicators of irreversible muscle degeneration. Future studies incorporating advanced quantitative sequences or serial imaging to track signal evolution would help differentiate transient changes from irreversible atrophy. Furthermore, future investigations should integrate quantitative strength testing (such as surface electromyography, Ito test, etc.) to fully characterize structure–function relationships in postoperative muscle recovery.

The results of paraspinal muscle CSA suggested that less paraspinal muscular injury in patients of Endo-LIF group than those of TLIF group at early follow up. Previous study revealed that atrophy of paraspinal muscles following TLIF procedure might be associated with direct muscle trauma during surgery, denervation and functional inactivation of fused segments [35]. The minimally invasive nature of Endo-LIF, characterized by smaller incisions (<  2 cm), limited vascular disruption, and preservation of myofascial attachments, may partially mitigate these detrimental processes through reduced soft-tissue insult.

Possible reasons for the observed discrepancy between PROs and imaging findings include several influencing factors on subjective reporting. Firstly, the ODI exhibits limited discriminatory ability at scores below 20%, making it challenging for patients to reliably differentiate between mild and moderate disability states or between clinically acceptable and completely normal function. Second, perceptual bias following the intervention may lead patients to report disproportionately elevated satisfaction and subjective scores based on even modest improvements. Third, the unrestricted use of non-steroidal anti-inflammatory drugs (NSAIDs) for analgesia in this study must be considered; while NSAIDs can alleviate pain and improve PROs via inhibition of the prostaglandin pathway, they do not modify the underlying pathology responsible for the persistent imaging manifestations observed in the paravertebral muscles.

### Limitations

Several important limitations warrant consideration. Firstly, the retrospective, non-randomized enrollment process was restricted to patients who underwent MRI examinations at short-term FU, potentially creating a non-representative cohort that could influence the observed associations. This study’s retrospective nature introduces potential selection bias, particularly in patient allocation to Endo-LIF vs. TLIF groups. While propensity score matching mitigated confounding, unmeasured variables (e.g., surgeon preference, unreported comorbidities) may persist. Secondly, the absence of long-term lumbar MRI images precluded assessment of temporal changes in paraspinal muscle morphology. Fat infiltration (irreversible degeneration biomarker), muscle quality metrics (signal normalization, perfusion), and functional assessments were not quantified, precluding differentiation between acute surgical injury and chronic degeneration. Thirdly, the non-randomized, single-center study's small sample size limits generalizability and statistical power, affecting conclusion reliability. The exclusion of surgical site infection patients limits generalizability to infected cohorts. Finally, the lack of daily serial monitoring prevented the accurate identification of peak inflammatory marker levels and the precise determination of the time course required for these values to return to baseline. Despite structured education and follow-up monitoring, heterogeneous patient adherence to home-based exercises may influence paraspinal muscle recovery. Collectively, these limitations underscore the preliminary nature of our observations. Future research under controlled conditions employing randomized designs, prospective longitudinal FU, multi-center recruitment for larger sample sizes, advanced imaging metrics and more intensive perioperative biomarker sampling, wearable sensors for objective compliance tracking is essential to validate and extend the implications of this work.

## Conclusion

Endo-LIF demonstrated reduced blood loss, shorter hospitalization and attenuated acute muscle injury markers versus TLIF. These advantages were offset by longer operative times and increased fluoroscopy. Both techniques achieved comparable long-term functional outcomes with similar safety profiles. Paraspinal muscle CSA findings indicated a lesser extent of early injury with Endo-LIF versus TLIF. Future validation of these structural changes might contribute to optimize postoperative rehabilitation strategies.

## Data Availability

No datasets were generated or analysed during the current study.

## Abbreviations

Endo-LIF: Endoscopic lumbar interbody fusion
TLIF: Transforaminal lumbar interbody fusion
LSS: Lumbar spinal stenosis
CT: Computed tomography
MRI: Magnetic resonance imaging
ODI: Oswestry disability index
VAS: Visual analogue scale
CK: Creatine kinase
CRP: C-reactive protein
ESR: Erythrocyte sedimentation rate
BMI: Body mass index
IQR: Inter quartile range
ICC: Intraclass correlation coefficient
IC: Confidence interval
M: Male
F: Female
BMD: Bone mineral density
ASA: American Society of Anaesthesiologist
TCSA: Total cross-sectional area of multifidus and erector muscle
FCSA: Functional cross-sectional area of multifidus and erector muscle
ES: Erector spinae
MF: Multifidus
ALIF: Anterior lumbar interbody fusion
CSF: Cerebrospinal fluid
